# Biosynthesis of Panaxynol and Panaxydol in *Panax ginseng*

**DOI:** 10.3390/molecules18077686

**Published:** 2013-07-02

**Authors:** Nihat Knispel, Elena Ostrozhenkova, Nicholas Schramek, Claudia Huber, Luis M. Peña-Rodríguez, Mercedes Bonfill, Javier Palazón, Gesine Wischmann, Rosa M. Cusidó, Wolfgang Eisenreich

**Affiliations:** 1 Lehrstuhl für Biochemie, Technische Universität München, Lichtenbergstrasse 4, 85747 Garching, Germany; 2 Laboratorio de Fisiología Vegetal, Facultad de Farmacia, Universidad de Barcelona, 08028 Barcelona, Spain; 3 FloraFarm, Bockhorn, 29664 Walsrode-Bockhorn, Germany

**Keywords:** *Panax ginseng*, Araliaceae, panaxynol, panaxydol, falcarinol, polyyne, crepenynic acid, isotopologue profiling, ^13^CO_2_

## Abstract

The natural formation of the bioactive C_17_-polyacetylenes (−)-(*R*)-panaxynol and panaxydol was analyzed by ^13^C-labeling experiments. For this purpose, plants of *Panax ginseng* were supplied with ^13^CO_2_ under field conditions or, alternatively, sterile root cultures of *P. ginseng* were supplemented with [U-^13^C_6_]glucose. The polyynes were isolated from the labeled roots or hairy root cultures, respectively, and analyzed by quantitative NMR spectroscopy. The same mixtures of eight doubly ^13^C-labeled isotopologues and one single labeled isotopologue were observed in the C_17_-polyacetylenes obtained from the two experiments. The polyketide-type labeling pattern is in line with the biosynthetic origin of the compounds via decarboxylation of fatty acids, probably of crepenynic acid. The ^13^C-study now provides experimental evidence for the biosynthesis of panaxynol and related polyacetylenes in *P. ginseng* under *in planta* conditions as well as in root cultures. The data also show that ^13^CO_2_ experiments under field conditions are useful to elucidate the biosynthetic pathways of metabolites, including those from roots.

## 1. Introduction

**Figure 1:**
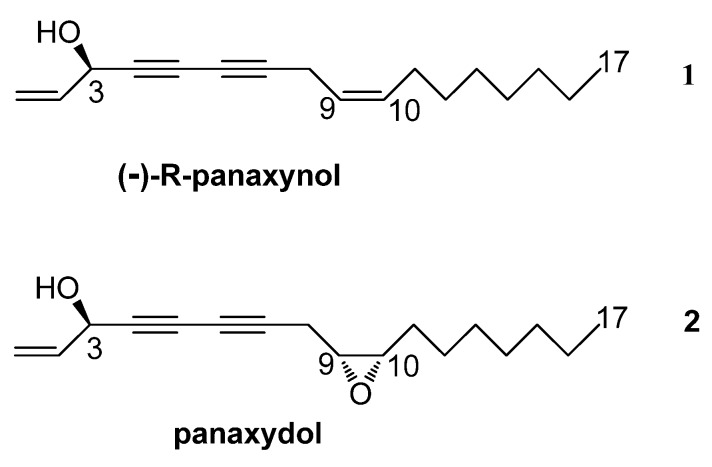
Structures of (−)-(*R*)-panaxynol (**1**) and panaxydol (**2**).

Extracts of ginseng (*Panax ginseng* C.A. Meyer) roots are used as health promoting drugs in traditional Oriental medicine. In recent times, however, ginseng has also gained importance in Western medicine as an anti-aging drug with an increasing market value [1]. Although the mechanisms of action of ginseng on human metabolism and health are not well understood, bioactivity is mainly assigned to the presence of ginsenosides, a group of secondary metabolites belonging to the triterpene saponins class [2,3,4]. However, additional bioactive natural products are present in the extracts of *P. ginseng* that contribute to the overall effect of ginseng. Among these bioactive metabolites, the C_17_-polyacetylenes, which include panaxynol (**1**, Figure 1) and its related epoxide panaxydol (**2**), have attracted remarkable interest mainly due to their biological activities [5]. Panaxynol was first isolated from roots of *P. ginseng* C.A. Meyer and described in 1964 [6]. To date, more than 16 polyacetylenes have been reported from *P. ginseng* [7] and other plants, mainly from the Araliaceae and Apiaceae families, including carrots, parsnip, parsley, fennel and celery [8,9]. 

Panaxynol and related polyynes have shown cytotoxic activity against several human tumour cell lines *in vitro* [10,11,12,13,14,15]. *In vivo* studies have confirmed the high potential of these metabolites for antitumour treatment [13]. Panaxynol-type polyacetylenes also exhibit significant antimicrobial (e.g., antimycobacterial) [16], antifungal [17,18,19], antiplatelet and anti-inflammatory [20,21,22,23], neuroprotective [24,25], antimutagenic [26,27,28], antiproliferative [12,21,29,30], antitrypanosomal [4], allergenic and skin-irritating activities [31,32,33,34]. The broad bioactivity of these metabolites, in combination with their high potential to benefit human health, reflects the importance of these polyacetylenes and the need for more detailed studies of their biosynthetic route, as a prerequisite to perhaps produce them by biotechnological means (e.g., using modified plants or recombinant microbial cultures).

On the basis of their structural similarity to fatty acids and of early experiments with radiolabeled fatty acids, it is widely accepted that the linear C_17_ polyacetylenes are derived from C_18_ unsaturated fatty acids [35,36,37] (reviewed in [9]). It has also been proposed, without experimental validation, that 3-hydroxyoleic acid could serve as an intermediate in panaxynol biosynthesis [36] and that aryl polyacetylenes are derived from the shikimate pathway [9]. However, the experimental evidence for the fatty acid route leading to C_17_ polyacetylenes is rather weak due to low incorporation rates of the radiolabeled precursors into the final products and the question remains open whether the fatty acid route is the main and only biosynthetic pathway leading to these secondary metabolites. In this study, we have used ^13^CO_2_ and ^13^C-labeled glucose as tracers for *in vivo* isotope labeling of *P. ginseng* plants and root cultures, respectively, to elucidate the biosynthetic pathway of C_17_ polyacetylenes.

## 2. Results and Discussion

### 2.1. Isolation and Identification of Panaxynol (1) and Panaxydol (2)

Lyophilized roots from plants treated with ^13^CO_2_ or root cultures enriched with [U-^13^C_6_]glucose were extracted with hexane. Purification of the corresponding extracts using column chromatography yielded the less polar panaxynol (**1**) and more polar panaxydol (**2**) in pure form; both metabolites were identified by comparing their spectroscopic data (^1^H and ^13^C-NMR) to those reported in the literature [38,39,40,41]. However, in view of the conflicting reports on the structures of this type of polyacetylenes (e.g., their stereoconfigurations), it is important to emphasize the correct identification of the isolated panaxynol; this metabolite was originally reported by Takahashi from *P. ginseng* [6] and later reported with the names falcarinol from *Falcaria vulgaris* [42] and carotatoxin from *Daucus carota* [43]. The first attempt to establish the absolute stereochemistry at C-3 of the compound was carried out by Larsen *et al.* [44], who described falcarinol from *Seseli gummiferum* as having a 3-(*R*) chirality on the basis of chemical correlation studies. The second attempt was carried out by Shim *et al.* [45,46] who described panaxynol as having a 3-(*S*) chirality on the basis of CD measurements. More recently, modified Mosher’s methods have described falcarinol from *Dendropanax arboreus* as being dextrorotatory and having the 3-(*S*) chirality [47], whereas panaxynol from *P. ginseng* was reported as being levorotatory and having the 3-(*R*) chirality [40]. These later reports were confirmed by Zheng *et al.* [48] who carried out the enantiospecific synthesis of the two isomers of falcarinol/panaxynol and demonstrated that the 3-(*R*) and 3-(*S*) chiralities correspond to the levorotatory and dextrorotatory enantiomers, respectively. The negative value of the optical activity of panaxynol obtained in this study also indicated its 3-(*R*) chirality.

### 2.2. Biosynthesis of Panaxynol and Panaxydol in P. ginseng

#### 2.2.1. *In planta* Experiments with ^13^CO_2_

Experiments with ^13^CO_2_ best resemble the physiological conditions for plants and the labeling profiles in the biosynthetic products represent *quasi* undisturbed *in planta* conditions*.* More specifically, the results obtained from these experiments are free from artifacts due to metabolic stress reactions (e.g., triggered by wounding in labeling experiments with cut plant organs) or due to the usage of non-physiological substrates in experiments with cell cultures. The strategic idea behind isotopologue profiling using ^13^CO_2_ is the photosynthetic generation of completely ^13^C-labeled metabolic intermediates (e.g., triose and pentose phosphates and products thereof) during an incubation period with ^13^CO_2_ (pulse period). During a subsequent chase period, the plants are allowed to grow under standard conditions (*i.e.*, in a natural atmosphere with ^12^CO_2_) for several days in which unlabeled photosynthetic intermediates are generated (*i.e.*, with ^12^C). These ^13^C- and ^12^C-intermediates from the pulse and the chase periods, respectively, are then taken by the plant as precursors for downstream biosynthetic processes. Consequently, the combination of these precursor units results in specific mixtures of ^13^C-isotopologues in the product. In other words, mixtures of unlabeled and multiple ^13^C-labeled isotopologues are generated as a consequence of the biosynthetic history of the metabolites under study. Using quantitative NMR spectroscopy, these isotopologue profiles can be assigned and attributed to biosynthetic pathways. Several recent examples have demonstrated the power of this experimental approach [49,50,51].

**Figure 2:**
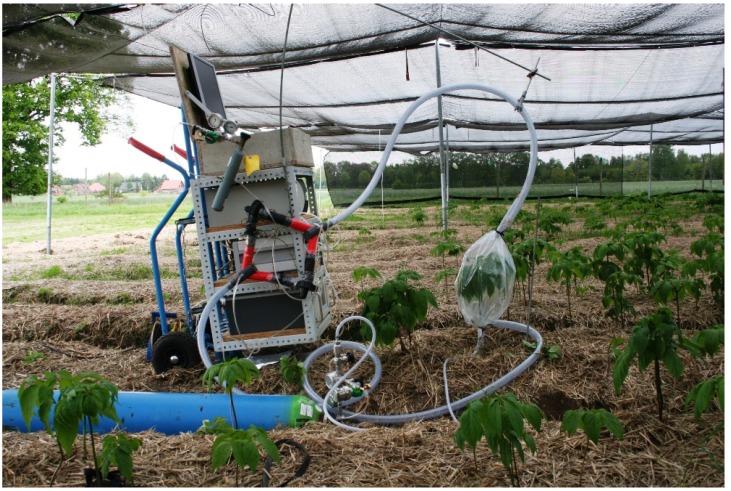
The portable unit used in this study for controlled incubation of *P. ginseng* with ^13^CO_2_ under field conditions.

**Figure 3:**
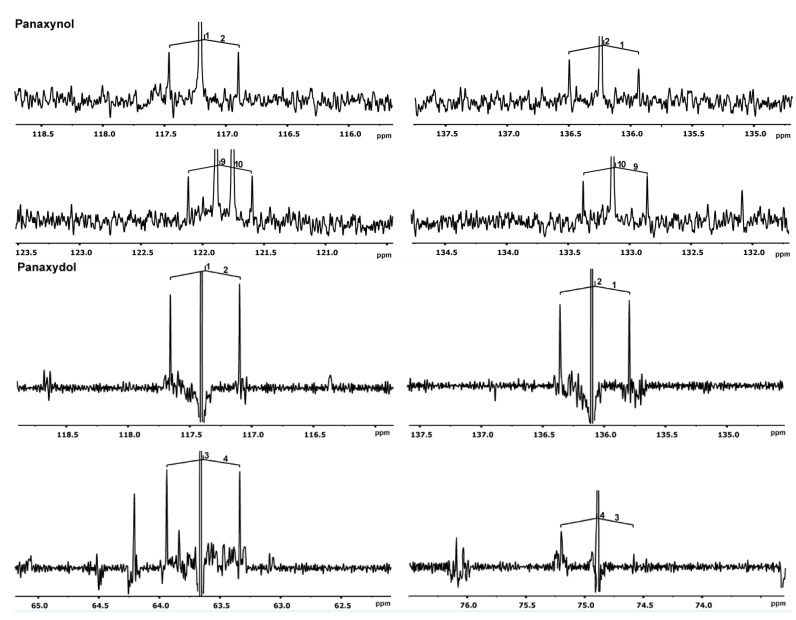
^13^C-NMR signals of panaxynol (**1**) and panaxydol (**2**) from the ^13^CO_2_ experiment. Couplings between ^13^C-atoms are indicated. Notably, satellites due to couplings between three adjacent ^13^C-atoms are not observed in the upfield or downfield regions of the doublets.

^13^CO_2_-labeling experiments of *P. ginseng* (Figure 2) were carried out using a portable ^13^CO_2_ unit [52]. To this aim, six-year-old plants of *P. ginseng* growing under field conditions were exposed to a ^13^CO_2_ atmosphere for 9.5 h and then allowed to grow for 19 days under natural conditions. Extraction of the roots yielded a mixture which, after purification, led to the isolation of labeled panaxynol (**1**) and panaxydol (**2**). The overall ^13^C-abundances (as determined from the ^1^H-NMR spectra of the compounds) were 1.5–2% for all carbon atoms. The relative intensities of the singlet signals due to ^13^C_1_-isotopologues in the ^13^C-NMR spectra of the labeled and unlabelled samples were identical. However, in the ^13^C-NMR spectra of labeled panaxynol (**1**) and panaxydol (**2**), all carbon signals, with the exception of the methyl carbon signals at 14.3 ppm, showed ^13^C-coupled satellite pairs (reflecting ^13^C_2_-isotopologues; Table 1, Table 2; Figure 3) at relative intensities of ca. 15% in the overall signal integrals for a given carbon atom. The same satellites from the unlabelled samples could display only 1% relative intensity in the global intensity of a given carbon due to the natural ^13^C-abundance of a ^13^C_2_-isotopologue (*i.e.*, 0.01 mol%). Notably, with the given amounts of the samples (*i.e.*, 2–3 mg, respectively), these natural abundance satellites could not be detected at all with the unlabelled compounds due to the low intrinsic sensitivity of ^13^C-NMR spectroscopy (for a review of quantitative ^13^C-NMR spectroscopy, see [53]). With the ^13^C-enriched samples, however, these satellites were detected and the analysis of the coupling constants for the ^13^C_2_-signals allowed the assignments of eight pairs of ^13^C_2_-labeled isotopologues, namely [1,2-13C2]-, [3,4-13C2]-, [5,6-13C2]-, [7,8-13C2]-, [9,10-13C2]-, [11,12-13C2]-, [13,14-13C2]-, [15,16-13C2]-**1** and -**2**, respectively, at similar or identical abundances of ca. 0.2 mol-% (see also Figure 4 where these isotopologues are indicated by bold bars connecting ^13^C-atoms in the molecule). The observed pattern of adjacent ^13^C-pairs indicates a polyketide-type biosynthesis, starting from [1,2-13C2]-acetyl-CoA/malonyl-CoA via a mixture of [1,2-13C2]-, [3,4-13C2]-, [5,6-13C2]-, [7,8-13C2]-, [9,10-13C2]-, [11,12-13C2]-, [13,14-13C2]-, [15,16-13C2]-, and [17,18-13C2]-fatty acids that is finally converted into the isotopologue profile of **1** and **2**. 

**Scheme 1:**
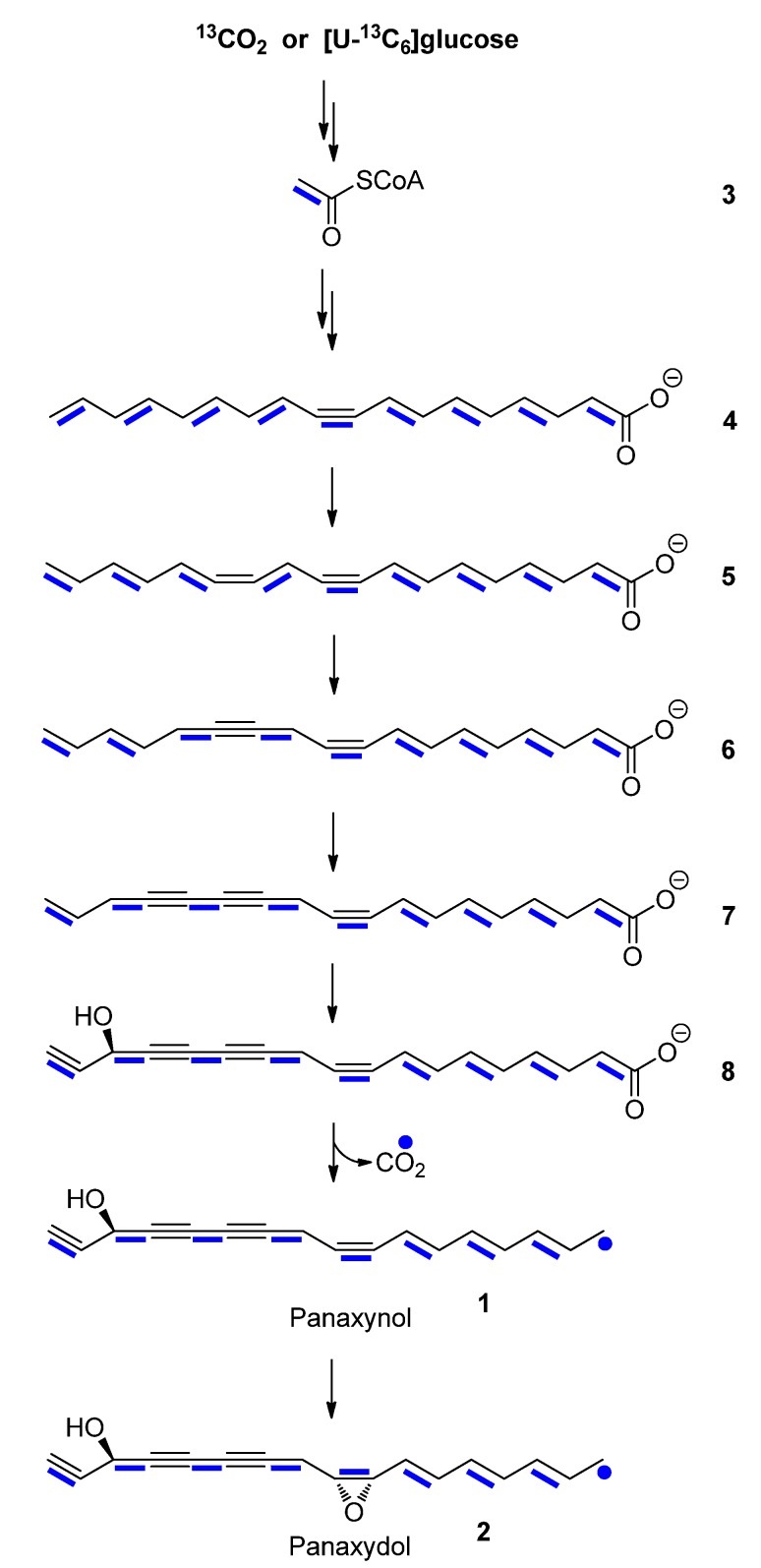
Proposed biosynthetic pathway of panaxynol (**1**) and panaxydol (**2**). Adjacent ^13^C-atoms detected in experiments with ^13^CO_2_ or [U-^13^C_6_]glucose are indicated by blue bars.

On this basis, it can be concluded that decarboxylation of a putative C_18_-intermediate takes place at the site where the uncoupled methyl group is finally observed in panaxynol or panaxydol (cf. Figure 4). Furthermore, it is suggestive to propose oleic acid (**4**) and crepenynic acid (**6**) as potential intermediates, since the triple and double bonds are located at the same positions as in the C_17_-polyacetylenes after decarboxylation. Decarboxylation could then occur at the level of intermediate **8** resulting in panaxynol (**1**) (Scheme 1). Panaxydol (**2**) could be formed by oxygenation of the C9-C10 double bond in panaxynol. However, it is important to keep in mind that the decarboxlation step can also occur upstream, *i.e.*, with 3-hydroxyoleic acid or 3-hydroxylinoleic acid as intermediates. However, in this scenario desaturases would unusually act on non-carboxylated substrates in order to introduce the required triple and double bonds in panaxynol and panaxydol. Theoretically, carboxylation of a labeled C_16_-fatty acid intermediate could also give rise of the detected labeling pattern. On the other hand, this hypothesis would be in contrast to earlier results that reported C_18_-precursors for C_17_-polyacetylenes [9,35,36,37].

**Table 1:** ^1^H- and ^13^C-NMR data of ^13^C-labelled panaxynol (**1**) (solvent, CDCl_3_; δ in ppm).

Atom	^1^H (δ)	J_HH_ (Hz)	Atom	^13^C (δ)	J_CC_ (Hz)
1a 1b 2 3 8a 8b 9 10 11 12 13 14 15 16 17	5.26 5.47 5.95 4.92 2.39 2.70 3.14 2.96 1.45–1.55 1.25–1.40 1.25–1.40 1.25–1.40 1.25–1.40 1.25–1.40 0.89	1H, ddd; 10.2, 1.5, 1.0 1H, ddd; 17.1, 1.5, 1.0 1H, ddd;16.8, 10.2, 5.4 1H, br d; 5.2 1H, ddd;17.7, 7.1, 0.9 1H, ddd; 17.7, 5.5, 0.9 1H, ddd; 7.1, 5.5, 4.2 1H, br td; 6.1, 4.1 2H, m 10H, m 10H, m 10H, m 10H, m 10H, m 3H, br t; 6.8	1 2 3 4 5 6 7 8 9 10 11 12 13 14 15 16 17	117.4 136.1 63.7 75.0 71.0 66.4 77.4 19.6 54.4 57.1 27.7 26.6 29.6 29.3 31.9 22.8 14.3	70.9 70.9 75.8 75.8 156.9 157.0 nd * 68.2 29.9 29.9 33.8 33.9 45.7 45.3 34.5 34.5 -

* nd, cannot be measured due to signal overlap.

**Table 2:** ^1^H- and ^13^C-NMR data of ^13^C-labelled panaxydol (**2**) (solvent, CDCl_3_; δ in ppm).

Atom	^1^H (δ)	J_HH_ (Hz)	Atom	^13^C (δ)	J_CC_ (Hz)
1a 1b 2 3 8 9 10 11 12 13 14 15 16 17	5.47 5.24 5.94 4.91 3.03 5.39 5.52 2.03 1.24–1.39 1.24–1.39 1.24–1.39 1.24–1.39 1.24–1.39 0.88	1H, ddd; 17.1, 1.2 1H, ddd; 10.1, 1.2 1H, ddd; 17.0, 10.2, 5.4 3H, t; 5.9 2H, d; 6.9 1H, ddddd; 11.3, 6.1, 1.6 1H, ddddd; 9.8, 8.1, 1.7 2H, ddd; 10.7, 6.9 10H, m 10H, m 10H, m 10H, m 10H, m 3H, t; 6.9	1 2 3 4 5 6 7 8 9 10 11 12 13 14 15 16 17	117.2 136.3 63.7 74.3 71.5 64.1 80.5 17.8 122.0 133.3 27.4 29.3 29.3 29.3 31.7 22.8 14.3	71.0 70.7 76.0 76.0 156.6 nd * 68.1 67.8 71.4 71.3 34.0 34.0 34.6 34.6 34.5 34.5 -

* nd, cannot be measured due to signal overlap.

#### 2.2.2. Experiments in Root Cultures with [U-^13^C_6_]glucose

Panaxynol (**1**), but not panaxydol (**2**), was obtained from the extract of *P. ginseng* hairy root cultures cultivated for four weeks in SH medium [54] containing a mixture of 88 mM sucrose and 4.4 mM [U-^13^C_6_]glucose. The carbon signals in the ^13^C-NMR spectrum of **1** showed the characteristic ^13^C-coupling satellites, with coupling constants identical to those described above for the panaxynol sample obtained from plants labeled with ^13^CO_2_ (Table 1). However, the relative sizes of the ^13^C-coupling satellites were higher (ca. 70% in the overall intensity for a given carbon) in the ^13^C spectrum of the panaxynol from the cultures than those from the field-grown plants. Not surprisingly, this reflects the high incorporation rates of [U-^13^C_6_]glucose in the stationary labeling experiment with the root cultures. It can be concluded that [U-^13^C_6_]glucose was efficiently incorporated into **1** via [U-^13^C_2_]acetyl-CoA following the same or closely related biosynthetic routes as described above for the plants growing under natural conditions. This shows that the pathways are obviously not affected by the supply of carbohydrates as carbon sources for root cultures. 

## 3. Experimental

### 3.1. Chemicals

^13^CO_2_ and [U-^13^C_6_]glucose (99% ^13^C-abundance) and other compounds were obtained from Sigma-Aldrich (Steinheim, Germany). 

### 3.2. Plants and Labeling Experiments with ^13^CO_2_

Labeling experiments with ^13^CO_2_ were carried out in August 2011 using six-year-old plants of *Panax ginseng* C.A. Meyer growing in the commercial field of FloraFarm (Walsrode, Germany). The plant was kept under a ^13^CO_2_ atmosphere (700 ppm) from 9 am for 9.5 h (pulse period; ^13^CO_2_ consumption: about 11 L) and left for 19 days (chase period) under natural field conditions before collecting the roots for extraction. The roots were washed, cut in pieces, frozen (liquid nitrogen) and lyophilized. Finally, the dry root pieces were ground using a mortar.

### 3.3. Root Cultures

Transformed roots of *P. ginseng* C.A. Meyer were induced from four-year-old rhizomes after infection with *Agrobacterium rhizogenes* A4 strain. Sterilized root discs were wounded with a sterile needle loaded with an *A. rhizogenes* suspension grown in liquid YEB medium [54] for 24 h at 28 ± 2 °C on a rotary shaker (Adolf Kühner AG, Birsfelden, Switzerland) at 100 rpm. The inoculated root discs were placed on Schenk and Hildebrandt’s medium (SH) [55] containing 3% (w/v) sucrose, 0.1% (w/v) myo-inositol and 0.27% (w/v) Phytagel (Sigma) at 26 °C. The medium was adjusted to pH 7.0 before autoclaving. After two days of co-cultivation, the explants were transferred to fresh medium containing cefotaxime (500 mg/L) in order to eliminate bacteria. After one or two months of cultivation, roots started to appear at the infection sites. In order to obtain the root lines, single roots were picked off and placed onto new media containing cefotaxime. Hairy roots free of bacterial contamination were cultured on hormone-free SH solid medium, in the dark at 26 °C. After six months of subculturing, the roots were cultured every two weeks on fresh solid medium and the selected root lines were transferred to SH liquid medium and kept in a rotary shaker at 100 rpm and 26 °C in the dark. The transformed nature of these root lines was confirmed by the presence of the TL-DNA rol C gene in the plant genome, detected by the pRiA4 by polymerase chain reaction analysis as described previously [56].

### 3.4. Labeling Experiments with [U-^13^C_6_]Glucose

The selected *P. ginseng* root line was cultured for four weeks in hormone-free SH liquid medium supplemented with 4.4 mM of [U-^13^C_6_]glucose. The root cultures were initiated from inocula of 2 ± 0.2 g of roots (fresh weight) maintained in 100-mL Erlenmeyer flasks, each containing 20 mL of SH medium. In this experiment, a total of 70 Erlenmeyer flasks were used, which corresponds to a total volume of 1.4 L. After four weeks, the roots (381 g, fresh weight) were harvested by filtration and freeze dried (21 g, dry weight). 

### 3.5. Isolation of Panaxynol (**1**) and Panaxydol (**2**)

Dry-powdered roots or root cultures (about 20 g) were extracted by refluxing (70 °C) twice for 3 h with hexane (300 mL). The solvent was evaporated under reduced pressure and the resulting crude extract (209 mg) was purified by open column chromatography (3 × 25 cm) using a mixture of hexane/acetone/methanol (80:18:2; v/v) as the eluting solvent (fraction volume, 5 mL). Panaxynol (**1**, 2.3 mg) was obtained in pure form at a retention volume of 155 mL. Panaxydol (**2**, 2.0 mg) was obtained in pure form from the field-grown roots of *P. ginseng* at a retention volume of 205 mL. The identity of both metabolites was confirmed by comparing their spectroscopic data (^1^H- and ^13^C-NMR) with those reported in literature [38,39,40,41]. The chirality at the C-3 position of panaxynol (**1**) was established as (*R*) by comparing its optical activity value ([α]_D_ −28.5°, c 0.17, CHCl_3_) with that reported in the literature ([α]_D_ −31.5°, c 1.0, CHCl_3_) [57].

### 3.6. Chromatography

Thin-layer chromatography (TLC) was carried out using aluminum-backed silica gel (60 F_254_) plates (Merck, 0.2 mm thickness) and the spots on the TLC plates were visualized by using a solution of H_2_SO_4_/MeOH (1:10; v/v) followed by heating (95–100 °C). Column chromatography purifications were performed using silica gel 60 (0.063–0.200 mm; 70–230 mesh; ASTM) from Merck (Darmstadt, Germany).

### 3.7. NMR Spectroscopy and Optical Rotation

NMR spectra were recorded at 27 °C using DRX 500, Avance I 500 and Avance III 500 spectrometers (Bruker Instruments, Karlsruhe, Germany). ^1^H- and ^13^C-NMR spectra were measured in CDCl_3_. For the measurement of ^13^C-NMR spectra, a cryo-probe head (5 mm QNP, inner coil = ^13^C) was used. One-dimensional ^1^H-spectra and COSY, HSQC, and HMBC experiments were performed with an inverse probe head (5 mm SEI, inner coil = ^1^H). The resonance frequencies of ^1^H and ^13^C were 500.1 MHz and 125.8 MHz respectively. Data analysis was done with TOPSPIN 3.0 (Bruker) or MestReNova 7.0.0 (Mestrelab Research, Santiago de Compostela, Spain). The optical rotation was measured using a Perkin Elmer 241 MC polarimeter (Perkin Elmer, Waltham, MA, USA). 

## 4. Conclusions

NMR-based isotopologue profiling of panaxydol and panaxynol confirmed their assumed origin from acetyl-CoA/malonyl-CoA via fatty acids with crepenynate as the putative intermediate. The decarboxylation site of the C_18_ intermediate(s) could now be clearly located to the methyl site of the product, panaxynol/panaxydol. The knowledge about the metabolite flux leading to the bioactive compounds and the factors for its control are useful to establish plants or root cultures of *P. ginseng* to produce C_17_-polyacetylenes at high yields. The study shows the feasibility of ^13^CO_2_-experiments to elucidate the biosynthetic origin of metabolites/products in field-grown plants. With the present study, it is demonstrated that the biosynthesis of root metabolites can be studied by pulse/chase experiments starting from the fixation of ^13^CO_2_ by the leaves.
